# Cold Stress Induces Tissue-Specific Lipid Metabolic Responses and Scd1-Mediated Hepatic Apoptosis in Silver Pomfret

**DOI:** 10.3390/ani16081196

**Published:** 2026-04-14

**Authors:** Man Zhang, Lu Zhang, Zi Yuan, Shengwei Xu, Yuguang Chen, Fangjun Xu, Yubei Qiu, Mengke Tang, Qinqin Dai, Yuanbo Li, Jiabao Hu, Yajun Wang

**Affiliations:** 1 Key Laboratory of Applied Marine Biotechnology, Ministry of Education, Ningbo University, Ningbo 315832, China; 2 College of Marine Sciences, Ningbo University, Ningbo 315211, China; 3 Conson Oceantec Valley Co., Ltd., Qingdao 266200, China; 4 Marine and Fisheries Research Institute, Ningbo 315103, China; 5 Jiangsu Innovation Center of Marine Bioresources, Nanjing 210019, China; 6 XiangShan (NingBo University) Aquatic Seed Industry Innovation Research Institute, Ningbo 315211, China

**Keywords:** *Pampus argenteus*, low-temperature stress, lipid metabolism, *scd1*, apoptosis

## Abstract

This study investigated the adaptive mechanisms of silver pomfret in response to chronic low-temperature stress, focusing on the tissue-specific expression patterns of the key lipid metabolism gene *scd1* and its central role in regulating hepatic apoptosis. A gradual cooling experiment (from 18 °C to 6 °C) was conducted to analyze the spatiotemporal expression profiles of ten lipid metabolism-related genes across six tissues. The results revealed that the most pronounced changes were observed in the heart, liver, and gills. The liver and heart rapidly activated genes involved in lipid breakdown and utilization from 16 to 12 °C for immediate energy supply, while gill tissue upregulated the *pi3k*/*p450*/*srebp*/*scd1* pathway at 10 °C to remodel membrane lipids against sustained stress. Further in vitro hepatocyte experiments demonstrated that *scd1* expression directly regulated cell survival and apoptosis under low temperatures. Knockdown of *scd1* significantly promoted apoptosis, whereas its overexpression effectively suppressed it. Moreover, *scd1* expression was intricately modulated by its upstream regulators *srebp* (positive regulation) and *pparγ* (showing potential negative feedback at specific temperatures). This study systematically elucidates the pivotal role of *scd1*-mediated lipid metabolic reprogramming in the cold adaptation of silver pomfret, providing a crucial theoretical foundation for deciphering the molecular mechanisms of cold tolerance and for breeding cold-resistant strains.

## Simple Summary

Silver pomfret is an important fish species, but it is sensitive to cold temperatures. In this study, we investigated how these fish adapt to long-term cold exposure by looking at changes in fat metabolism across different body tissues. We found that during cold stress, different tissues take on specific roles at different times. For example, the liver and heart quickly use stored fats for energy in the early stages, while the gills adjust their cell membranes to remain flexible later on. We also discovered that the *scd1* gene, involved in fat processing, plays a key role in protecting liver cells from dying under cold conditions. When this gene was active, cells survived better; when it was blocked, more cells died. Our findings help explain how fish cope with cold environments and may support future efforts to breed more cold-tolerant fish for aquaculture.

## 1. Introduction

Temperature is a critical environmental factor for fish, as ectothermic animals rely on external heat exchange to regulate physiological processes [1,2]. Fish inhabit diverse thermal environments and have evolved distinct adaptive strategies [3,4]. Based on thermal tolerance, they are classified as eurythermal, with broad temperature adaptability, or stenothermal, restricted to narrow temperature ranges [5,6]. Temperature influences key aspects of fish biology, including behavior, reproduction, and distribution [7,8]. Low-temperature stress, resulting from seasonal changes or sudden drops, can trigger physiological and biochemical responses that affect fish survival [9,10,11,12,13]. However, prolonged exposure leads to oxidative stress, causing cellular damage [14]. To adapt, fish modulate fatty acid composition, increasing unsaturated fatty acids to enhance membrane fluidity [15]. A key enzyme in this process is stearoyl-CoA desaturase-1 (SCD1), which is an enzyme localized to the endoplasmic reticulum. Its primary role is to convert saturated fatty acids (SFAs) into monounsaturated fatty acids (MUFAs), thereby regulating the saturation level of phospholipids in cell membranes, which crucially influences membrane fluidity and permeability. Notably, the described response patterns are typical for warm-water fish, whereas cold-water fish may exhibit different adaptive strategies. The *scd1* gene is evolutionarily conserved across fish species, with studies in large yellow croaker (*Larimichthys crocea*) and yellow catfish (*Pelteobagrus fulvidraco*) revealing its structural and functional similarities [16]. In silver pomfret (*Pampus argenteus*), *scd1* shares 90% sequence identity with gilthead sea bream (*Sparus aurata*), and its expression is regulated by *pparγ* (Peroxisome proliferator-activated receptor gamma) and *srebp1* (Sterol regulatory element-binding protein 1) [17]. While *scd1* expression increases in response to short-term cold exposure, its dynamics during long-term adaptation remain unclear. Furthermore, the molecular mechanisms and regulatory networks of *scd1* under low-temperature stress, as well as its impact on cellular apoptosis, are still poorly understood.

The silver pomfret, an economically important marine fish in China, has seen significant advancements in aquaculture, with breakthroughs in large-scale breeding achieved in 2016. The optimal temperature range for silver pomfret is 18–28 °C [18,19]. However, natural overwintering remains a challenge in northern farming regions, hindering industrial development. Increasingly frequent extreme winter temperatures further exacerbate these challenges [20]. To address this, our research group has initiated low-temperature breeding programs and conducted transcriptomic and preliminary molecular mechanism studies on the cold tolerance of silver pomfret [19,21]. However, the mechanisms underlying low-temperature adaptation in silver pomfret remain poorly understood.

This study investigates the spatiotemporal expression patterns of *scd1* (Stearoyl-CoA desaturase 1) and related lipid metabolism genes, including *pi3k* (Phosphoinositide 3-kinase), *P450* (Cytochrome P450), *cact* (Carnitine-acylcarnitine translocase), *srebp* (Sterol regulatory element-binding protein), *pparγ* (Peroxisome proliferator-activated receptor gamma), *pparα* (Peroxisome proliferator-activated receptor alpha), *stc* (Stanniocalcin), *mct* (Monocarboxylate transporter), and *ltp* (Lipid transfer protein) to elucidate their roles in six tissues: liver, kidney, gill, heart, brain, and muscle. By analyzing these genes and proteins, we aim to uncover the critical role of lipid metabolism in cold adaptation. The identified key genes, particularly *scd1* and its upstream regulators, have the potential to serve as molecular markers for cold tolerance, providing valuable targets for marker-assisted selection in low-temperature breeding programs. Additionally, this study explores the functional role of *scd1* in response to low-temperature stress, particularly its impact on hepatic apoptosis. Understanding how *scd1* influences liver cell survival under cold stress will provide further insights into the molecular mechanisms of low-temperature adaptation in silver pomfret, contributing to the development of targeted strategies—such as genetic improvement of cold-tolerant strains or the application of functional feed additives—to mitigate cold-induced damage in aquaculture.

## 2. Materials and Methods

### 2.1. Ethical Statement

All experimental procedures in this study complied with the China Government Principles for the Utilization and Care of Vertebrate Animals Used in Testing, Research, and Training (State Science and Technology Commission of the People’s Republic of China No. 2, 31 October 1988; http://www.gov.cn/gongbao/content/2011/content_1860757.htm, accessed on 10 October 2024). Ethical approval was obtained from the Institutional Animal Care and Use Committee of Ningbo University (Approval No. NBU20220079).

### 2.2. Experimental Design and Fish Culturing

A total of 600 six-month-old silver pomfret, with an average body length of 8.51 ± 0.54 cm and an average body weight of 25.59 ± 3.16 g, were used in the experiment. The selected size represents the juvenile stage of silver pomfret, which is a critical period for growth and development and is also the stage most susceptible to low-temperature stress in aquaculture. Moreover, our previous studies have shown that this size exhibits the most typical physiological responses to cold stress, making it suitable for mechanistic studies.

After a two-week acclimation period, the fish were evenly distributed into six tanks, with 100 individuals per tank as parallel experimental groups. During the experiment, the fish were fed HaiTong 8 floating juvenile feed three times daily, with a daily feeding amount equivalent to 2% of their body weight.

The cooling experiment was conducted in a recirculating aquaculture system at the pilot base of Ningbo University. Water quality parameters were maintained as follows: dissolved oxygen, 8.95 mg/L; salinity, 22 ± 1 ‰; pH, 8.16; nitrite, 0.327 ± 0.012 mg/L; ammonia nitrogen, 2.43 ± 0.03 mg/L; and a photoperiod of 12 h light and 12 h dark (12L:12D). The low-temperature stress experiment began at 18 °C, with a cooling rate of 0.5 °C per day. This temperature range was conditionally divided into three cooling phases: early (16–12 °C), middle (12–10 °C), and late (8–6 °C). Samples of heart, brain, muscle, liver, kidney, and gill tissues were collected at 18 °C, 16 °C, 14 °C, 12 °C, 10 °C, 8 °C, and 6 °C, with six fish collected at each temperature point. For each tissue, three biological replicates and three technical replicates were prepared, and all samples were immediately preserved in liquid nitrogen for subsequent quantification of lipid metabolism-related genes and proteins. The experimental design and sampling points are shown in Figure S1A.

### 2.3. Real-Time Quantitative PCR (RT-qPCR)

The extraction of total RNA was based on the Trizol method. Qualified RNA was used for genomic DNA removal and reverse transcription using the HiScript^®^ III All-in-one RT SuperMix Perfect for qPCR kit (Vazyme, Nanjing, China). The reaction mixture was prepared as follows: RNase-free ddH_2_O was added to a final volume of 20 µL, 4 µL of 5x All-in-one qRT SuperMix, 1 µL of Enzyme Mix, and template RNA (ranging from 1 pg to 1 µg of total RNA). The reaction program consisted of incubation at 50 °C for 15 min, followed by heat inactivation at 85 °C for 5 s. The resulting cDNA was either used immediately for qPCR or stored at −20 °C for future use.

Differentially expressed genes (DEGs) with *p* < 0.05 and |log2foldchange| > 2 were selected from the transcriptome data. Ten DEGs were chosen based on our previous transcriptome data (NCBI Accession Number: PRJAN783750) for validation using RT-qPCR. The cDNA served as the template, and *β-actin* was used as the reference gene. *β-actin* was selected based on its high amplification efficiency (99.6–101.2%) and stable expression, as determined by the Normfinder software (v0.953) among three candidate reference genes. The relative expression levels of DEGs were quantified using the 2^−ΔΔCt^ method, with 18 °C (the initial temperature of the experiment) as the control group, to validate the transcriptome results. Gene-specific primers were designed using Primer 5.0 software based on the transcriptome sequences. The primers were validated for size and specificity by PCR and gel electrophoresis, and their amplification efficiency (90–110%) was confirmed by RT-qPCR. The primer sequences are listed in Table 1.

### 2.4. Protein Extraction and Western Blot Analysis

Protein extraction was performed using RIPA lysis buffer (Solarbio, Beijing, China) supplemented with 1 mM PMSF protease inhibitor (Solarbio, China). Briefly, 20 mg of tissue was homogenized in 200 µL of lysis buffer on ice for 3 min using an electric homogenizer. The homogenate was incubated on ice for 30 min with vortexing every 10 min, followed by centrifugation at 12,000 rpm for 10 min at 4 °C. The supernatant was collected, and protein concentration was determined using the Kangwei BCA Protein Assay Kit (Kangwei Century, Taizhou, China) according to the manufacturer’s instructions. Protein samples were mixed with 5X SDS loading buffer (Beyotime, Shanghai, China) at a 1:4 ratio and boiled at 105 °C for 7 min before storage at −40 °C.

For Western blot analysis, 12.5% SDS-PAGE gels were prepared using the ExpressCast PAGE Color Gel Kit (New Cell & Molecular, Suzhou, China). Protein samples (20 µg per lane) and a protein marker (10–180 kDa, Solarbio, China) were loaded and electrophoresed at 150 V for 40–60 min. Proteins were transferred to PVDF membranes (Solarbio, China) using a semi-dry transfer system at 400 mA for 35 min. Membranes were blocked with rapid blocking buffer (New Cell & Molecular, China) for 15 min and incubated with primary antibodies (β-actin, 1:2000; Scd1, 1:1000; ABclonal, Wuhan, China) diluted in antibody dilution buffer (Beyotime, China) for 2 h at room temperature or overnight at 4 °C. After washing with TBST (Solarbio, China), membranes were incubated with HRP-conjugated secondary antibodies (1:2000; ABclonal, China) for 30 min. Protein bands were visualized using a chemiluminescence kit (Advansta, San Jose, CA, USA) and imaged with a gel documentation system. Band intensities were quantified using ImageJ software (https://ij.imjoy.io/, accessed on 13 November 2024).

### 2.5. Establishment of a Low-Temperature Model for Pampus argenteus Hepatocytes

The *Pampus argenteus* hepatocyte cell line [22] was established following the methodology described in our previously published article; the optimal culture temperature for PaL is 26 °C [23]. Hepatocytes were detached using TrypLE™ Express enzyme (Gibco, Waltham, MA, USA) and subsequently washed with complete medium. The cell suspension was centrifuged at 1000 rpm for 5 min. After cell collection, the hepatocytes were seeded in 96-well plates (Corning, New York, NY, USA) at a density of 10^3^ cells per well. Following 12 h of incubation at 2 °C to allow cell attachment, the cultures were transferred to incubators set at different temperatures: 26 °C, 18 °C, 10 °C, and 8 °C. Cell viability was assessed using the CCK-8 assay kit (Dojindo, Kumamoto, Japan) at three time points: 0 h, 24 h, and 48 h post-temperature treatment. Each experimental group included five replicates to ensure statistical reliability (Figure S1B).

### 2.6. RNAi of scd1 and Its Regulators (srebp/pparγ)

Three siRNA sequences were designed for each target gene, including *scd1* and its upstream regulators *srebp* and *pparγ* (Table 2). The siRNAs were transfected into *Pampus argenteus* hepatocytes using a lipid-based transfection reagent at a ratio of 1.5:1 (siRNA:liposome) based on our previous study [24]. The most effective siRNA for each gene was selected based on its maximal knockdown efficiency, as determined by RT-qPCR analysis. The detailed experimental procedures were performed according to the established protocol described by Zhang et al. [19].

### 2.7. scd1 Expression Plasmid Construction and Cellular Transfection

The open reading frame of *scd1* was amplified using primers containing homologous arms (F primer 5′–3′: ggatcccatcgattcgaattcATGACAGAGGCGGAGGCG, R primer 5′–3′: ggctcgagaggccttga attc TTATCAAAAAACAAAGTACGGACCA) and subsequently cloned into the pEASY^®^-T&B Zero Cloning vector (TransGen, Beijing, China). The resulting DNA fragment was then ligated into EcoR I (Takala, Tokyo, Japan)-digested pCS^2+^ vectors (Invitrogen, USA) to generate expression constructs pCS^2+^-*scd1*. Plasmid preparation was performed using the NovoRec^®^ plus One-step PCR Cloning Kit (Novoprotein, Suzhou, China). For cell transfection, a mixture containing 2500 ng plasmid DNA, 7.5 μL Lipofectamine 3000, and 10 μL p3000 reagent (Invitrogen, Carlsbad, CA, USA) was prepared for each well of a six-well plate. Transfection efficiency was monitored using the pCS-EGFP plasmid (Invitrogen, USA) and quantified by counting GFP-positive cells (*n* = 20).

### 2.8. Apoptosis Detection by TUNEL Assay

Apoptosis was assessed using the Terminal deoxynucleotidyl transferase dUTP nick end labeling (TUNEL) method. Tissue samples were processed following standard histological protocols for collection, fixation, embedding, and sectioning. After deparaffinization in xylene and rehydration through a graded ethanol series, sections were subjected to proteinase K digestion (Servicebio Technology Co., Ltd., Wuhan, China) at 37 °C for 25 min. Following three washes with phosphate-buffered saline (PBS, pH 7.4), tissue sections were incubated with the One-Step TUNEL Apoptosis Assay Kit (Beyotime Biotechnology, Shanghai, China) according to the manufacturer’s protocol. The reaction was carried out in a humidified chamber at 3 °C for 2 h. After additional PBS washes, nuclei were counterstained with DAPI at room temperature for 10 min. Finally, the sections were mounted and visualized using a Leica SP8 confocal microscope (Wetzlar, Germany).

### 2.9. Statistical Analysis

All experimental data were presented as mean ± standard deviation (mean ± SD). Statistical analyses were performed using one-way ANOVA in SPSS 20.0 software (IBM, Armonk, NY, USA). Data visualization was conducted using GraphPad Prism 8 (GraphPad Software, La Jolla, CA, USA). Statistical significance was determined at two levels: *p* < 0.05 was considered statistically significant, while *p* < 0.01 indicated highly significant differences.

## 3. Results

### 3.1. Spatiotemporal Expression Patterns of Lipid Metabolism-Related Genes and Proteins

**Figure 1:**
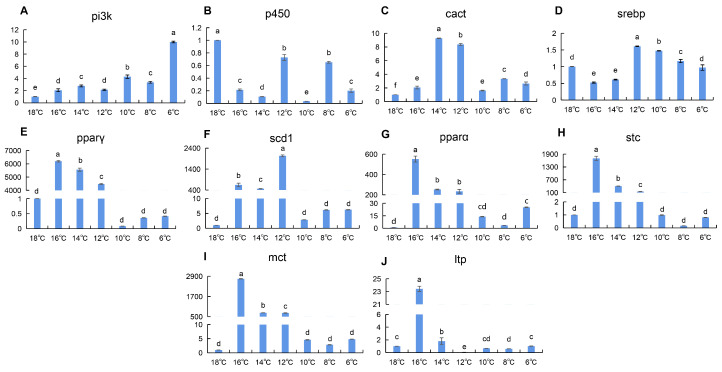
(**A**–**J**): Changes in relative expression of lipid metabolism genes in silver pomfret liver during cooling process (*n* = 3). X-axis = Temperature; Y-axis = Fold change (2^−ΔΔCt^). In the bar chart, lowercase letters denote statistical significance. Bars sharing the same letter are not significantly different, whereas those with different letters are significantly different.

Spatiotemporal quantification of 10 lipid metabolism-related genes revealed that the liver of silver pomfret exhibited the most rapid fatty acid mobilization during gradual cooling. In the early cooling phase (16–12 °C), the expression levels of *cact*, *pparγ*, *scd1*, *pparα*, *stc*, and *mct* were significantly upregulated compared to the control group (18 °C). However, in the late cooling phase, these genes were sharply downregulated (Figure 1C,E–I). Hepatic *pi3k* levels negatively correlated with temperature (Figure 1A), while *p450* was persistently suppressed (Figure 1B). *Srebp* showed delayed induction, increasing only in late cooling (Figure 1D). Additionally, the *ltp* gene showed significantly upregulated expression at 16 °C (Figure 1J).

**Figure 2:**
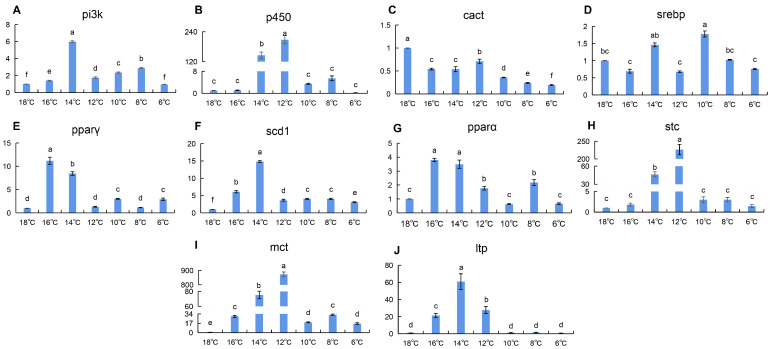
(**A**–**J**): Changes in relative expression of lipid metabolism genes in silver pomfret heart during cooling process (*n* = 3). X-axis = Temperature; Y-axis = Fold change (2^−ΔΔCt^). In the bar chart, lowercase letters denote statistical significance. Bars sharing the same letter are not significantly different, whereas those with different letters are significantly different.

During cooling, the expression of lipid metabolism genes in the heart of silver pomfret exhibited an initial increase followed by a subsequent decrease (Figure 2). Specifically, *p450*, *stc*, and *mct* peaked at 12 °C, while *pi3k*, *scd1*, *pparα*, and *ltp* reached maximal expression at 14 °C. In contrast, *cact* expression remained significantly lower than that of the control group throughout low-temperature exposure. *srebp* expression showed transient elevations at 14 °C and 10 °C but did not significantly differ from controls at other temperatures. Notably, *pparα* and *pparγ* both increased significantly at 16 °C and 14 °C. When the water temperature drops below 12 °C, the expression levels of most lipid metabolism-related genes rapidly decline. However, *srebp* and *pparα* exhibit distinct patterns: *srebp* shows a second upregulation at 10 °C, while *pparα* displays a notable upregulation at 8 °C.

**Figure 3:**
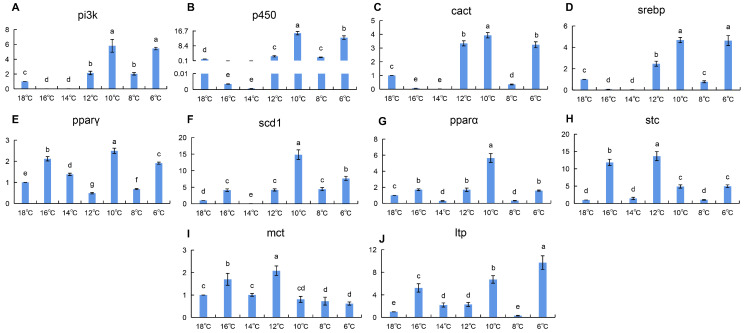
(**A**–**J**): Changes in relative expression of lipid metabolism genes in silver pomfret gill during cooling process (*n* = 3). X-axis = Temperature; Y-axis = Fold change (2^−ΔΔCt^). In the bar chart, lowercase letters denote statistical significance. Bars sharing the same letter are not significantly different, whereas those with different letters are significantly different.

The gill is not the primary organ for lipid metabolism. According to the RT-qPCR results (Figure 3), the expression levels of most lipid metabolism genes including *pi3k*, *p450*, *cact*, *srebp*, *pparγ*, *scd1*, *pparα*, and *ltp* were significantly upregulated at both 10 °C and 6 °C. However, unlike the liver tissue, where expression increased by several thousand-fold, the upregulation in gill tissue was only approximately tenfold compared to the control group.

In muscle tissue, most lipid metabolism-related genes (*pi3k, p450*, *cact*, *srebp*, *pparγ*, *pparα*, and *stc*) showed two distinct upregulation events during cooling, occurring around 14 °C and 6 °C, whereas scd1 and *pparα* exhibited a sharp increase when the temperature dropped below 8 °C. Meanwhile, mct expression remained consistently lower than that of the control group, and ltp showed no clear expression pattern (Figure S2).

In the kidney, the expression of most genes (*pi3k*, *p450*, *cact*, *srebp*, *scd1*, *pparα*, *stc*, and *ltp*) increased significantly during the later stages of cooling, with *pi3k*, *p450*, *cact*, and *srebp* peaking at 6 °C. In contrast, *pparγ* and *mct* showed significantly decreased expression throughout the cooling period (Figure S3).

In brain tissue, the expression of lipid metabolism-related genes did not correlate significantly with temperature changes. Notably, three genes (*pparα*, *pparγ*, and *scd1*) showed peak expression at 16 °C, while eight genes (*pi3k*, *p450*, *cact*, *srebp*, *pparγ*, *scd1*, *stc*, and *ltp*) peaked at 6 °C, indicating activation during both the early and final stages of the cooling period (Figure S4).

**Figure 4:**
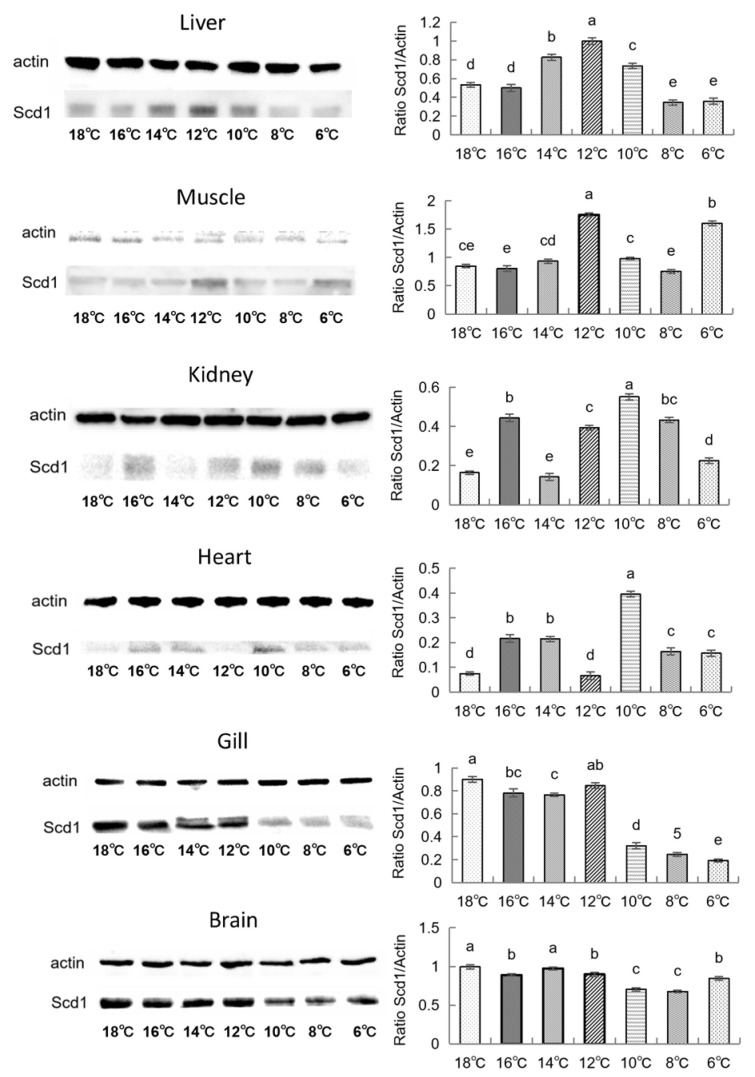
Changes in Scd1 protein expression across different tissues (*n* = 3). In the bar chart, lowercase letters denote statistical significance. Bars sharing the same letter are not significantly different, whereas those with different letters are significantly different.

Figure 4 shows the expression of Scd1 protein across six tissues of silver pomfret under low-temperature stress. The left panel displays the Western blot (WB) bands, while the right panel presents the relative expression levels (Scd1/Actin) calculated as grayscale values using ImageJ software (https://ij.imjoy.io/). The results indicate that the trends in Scd1 gene and protein expression were generally consistent in most tissues. However, protein expression lagged behind gene expression in muscle and heart tissue. As the temperature decreased, protein expression significantly decreased in the brain and gills compared to the control group. Notably, kidney and heart tissues showed significantly high protein expression during the early cooling phase, whereas liver and muscle tissues exhibited significantly high expression during the mid-to-late cooling phases.

### 3.2. Establishment of a Hypothermic Hepatocyte Model and Screening with siRNA

**Figure 5:**
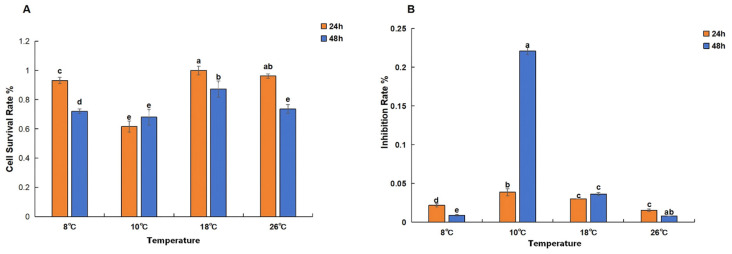
Viability (**A**) and inhibition rate (**B**) of silver pomfret hepatocytes at different temperatures (*n* = 3). In the bar chart, lowercase letters denote statistical significance. Bars sharing the same letter are not significantly different, whereas those with different letters are significantly different.

The effects of different temperatures and treatment durations on the viability of silver pomfret hepatocytes are shown in Figure 5A. When treated for 24 h, cell viability was highest at 18 °C, reaching 100%. This was followed by 26 °C, where cell viability was 96.2%; no significant difference was observed between 18 °C and 26 °C at 24 h. At 8 °C, cell viability decreased significantly compared to 26 °C, to 93% (*p* < 0.05). After 24 h at 10 °C, cell viability was the lowest among all groups, at 61.6%, which was significantly lower than that at other temperatures. After 48 h of treatment, cell viability in all temperature groups was significantly lower than that at 24 h (except for the 10 °C group, which showed no significant difference between 24 h and 48 h). The highest viability at 48 h was observed in the 18 °C group (87.2%). Cell viabilities at 8 °C, 10 °C, and 26 °C after 48 h were similar, at 73.7%, 68%, and 72%, respectively.

The inhibition rates of silver pomfret hepatocytes under different temperatures are shown in Figure 5B. Comparing the 24 h and 48 h groups at 8 °C, 10 °C, 18 °C, and 26 °C, the highest inhibition rate was observed after 48 h at 10 °C, reaching 22.1%. The inhibition rates in all other groups were below 5%. No statistically significant difference was found between the inhibition rates at 18 °C for 24 h and 48 h, which were 3% and 3.6%, respectively.

**Figure 6:**
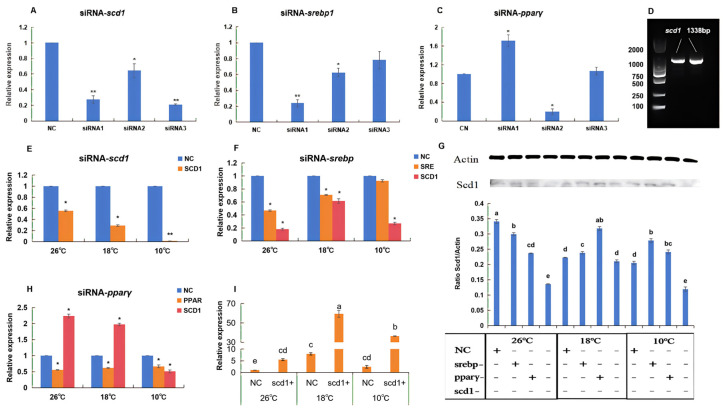
Expression changes in related genes and proteins in silver pomfret hepatocytes at different temperatures after gene knockdown or overexpression (*n* = 3). (**A**–**C**) Screening of siRNA knockdown efficiency, (**D**) the cloning results of the silver pomfret *scd1* gene, (**E**) *scd1* knockdown, (**F**) *srebp* knockdown, (**G**) resultant Scd1 protein levels. (**H**) *pparγ* knockdown, (**I**) *scd1* overexpression. In the bar chart, lowercase letters denote statistical significance. Bars sharing the same letter are not significantly different, whereas those with different letters are significantly different. * indicates a significant difference compared with NC (*p* < 0.05), and ** indicates a highly significant difference compared with NC (*p* < 0.01).

Figure 6A–C presents the screening results for siRNA interference efficiency in silver pomfret. The interference efficiency was evaluated based on the relative expression levels of the target genes (y-axis). The results indicated that siRNA3-*scd1*, siRNA1-*srebp*, and siRNA2-*pparγ* exhibited the highest interference efficiencies at 80.1%, 76%, and 80%, respectively, and were therefore selected for subsequent experiments. The cloning results of the silver pomfret *scd1* gene are shown in Figure 6D, with a full length of 1338 bp. Sequence alignment of the pCS^2+^ homologous recombination overexpression vector is presented in Figure S5. The coding region spans 1002 bp and is consistent with the silver pomfret *scd1* gene sequence (MK215078) published in NCBI, with only a few base mutations that do not affect subsequent experiments.

### 3.3. Functional Analysis of scd1 and Its Upstream Regulators Under Cold Stress

As shown in Figure 6E, the gene expression of *scd1* in silver pomfret hepatocytes was analyzed at different temperatures following its knockdown. After transfection, cells were cultured at different temperatures for 24 h. The expression level of *scd1* after knockdown at each temperature was compared relative to its respective normalized negative control (NC) group at the same temperature. The results showed that knockdown successfully reduced *scd1* expression, and the extent of reduction varied with temperature. Notably, the expression level was lowest at 10 °C, reaching only 0.0012 times that of the NC group.

To investigate the influence of two upstream regulators of *scd1*, *srebp* and *pparγ*, on *scd1* expression under low temperatures, we performed knockdown of these genes and analyzed the expression of both the targeted gene and downstream *scd1*. The results indicated that in silver pomfret, *srebp* acts as an upstream gene of *scd1*. After *srebp* knockdown, its own expression showed an inverse relationship with temperature. At 26 °C and 18 °C, *srebp* expression was significantly lower than in the NC group (*p* < 0.05), while no significant difference was observed at 10 °C. The expression of the downstream gene *scd1* following *srebp* knockdown was significantly lower than in the NC group at all temperatures (*p* < 0.05), specifically 0.18-fold at 26 °C, 0.62-fold at 18 °C, and 0.27-fold at 10 °C (Figure 6F).

In contrast, *pparγ* expression after its knockdown showed no clear correlation with temperature. However, the expression of downstream *scd1* following *pparγ* knockdown was significantly higher than in the NC group at 26 °C and 18 °C. At 10 °C, *scd1* expression was significantly lower, at 0.51-fold of the NC group (Figure 6G).

Following *scd1* overexpression in silver pomfret hepatocytes and 24 h of culture at different temperatures, its expression levels are shown in Figure 6H. Using the 26 –NC group as the reference control, *scd1* expression was significantly higher in all temperature groups (*p* < 0.05). The highest expression occurred at 18 °C, reaching 59.29-fold that of the control group.

Changes in Scd1 protein levels after knockdown of *srebp*, *pparγ*, or *scd1* and 24 h of culture at different temperatures are presented in Figure 6I. The results demonstrated that *srebp* and *pparγ* knockdown affected Scd1 protein expression at 26 °C but not at 18 °C or 10 °C. In contrast, direct knockdown of *scd1* significantly reduced Scd1 protein levels at both 26 °C and 10 °C (*p* < 0.05).

### 3.4. Effects of rnai and Overexpression on Cell Apoptosis Under Low Temperature Conditions

**Figure 7:**
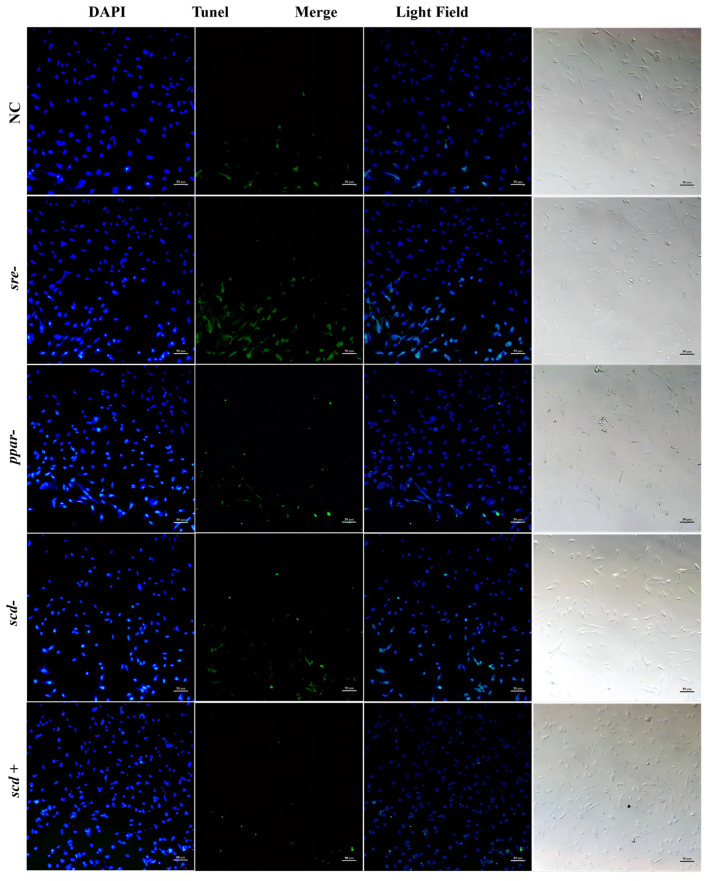
Apoptosis in silver pomfret hepatocytes after gene knockdown or overexpression at 26 °C (TUNEL-DAPI double staining, 10×).

Figure 7 shows the cell apoptosis observed at 26 °C after RNAi targeting *srebp*, *pparγ*, and *scd1*, as well as after *scd1* overexpression. Green fluorescence signals represent TUNEL-positive apoptotic cells, while blue signals indicate DAPI-stained nuclei. At 26 °C, the NC group showed a low level of apoptosis, accounting for approximately 15% of the total cells. The RNAi-*srebp* group exhibited the strongest apoptotic signals among all groups, with about 40% of cells being TUNEL-positive. The fluorescence intensity of apoptotic signals in the RNAi-*pparγ* and RNAi-*scd1* groups was similar to that in the NC group, each representing about 10% of total cells. In contrast, the *scd1* overexpression group showed the weakest green fluorescence, indicating the lowest level of apoptosis, at approximately 5% of total cells.

**Figure 8:**
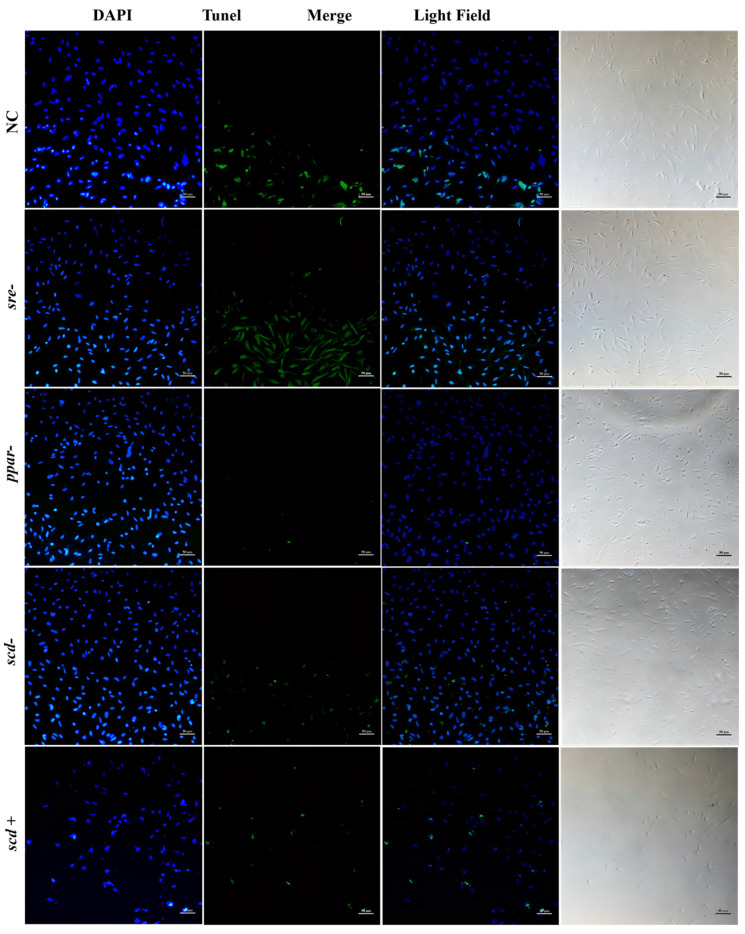
Apoptosis in silver pomfret hepatocytes after gene knockdown or overexpression at 18 °C (TUNEL-DAPI double staining, 10×).

Figure 8 illustrates apoptosis under the same treatments at 18 °C. Compared with 26 °C, the NC group displayed significantly stronger green fluorescence at 18 °C, reflecting increased apoptosis (~35% of total cells). The RNAi-*srebp* group showed even stronger fluorescence intensity, representing the most severe apoptosis at this temperature (~50% of total cells), which was significantly higher than that in the NC group. In contrast, the RNAi-*pparγ* group exhibited the weakest green fluorescence, with almost no detectable apoptosis (~1% of total cells), significantly lower than that of the NC group at the same temperature. The RNAi-*scd1* group also showed low fluorescence intensity, accounting for about 5% of total cells, significantly lower than that of the NC group. In the *scd1* overexpression group, the number of cells decreased (reduced blue fluorescence), and apoptotic signals remained weak, representing about 10% of total cells.

**Figure 9:**
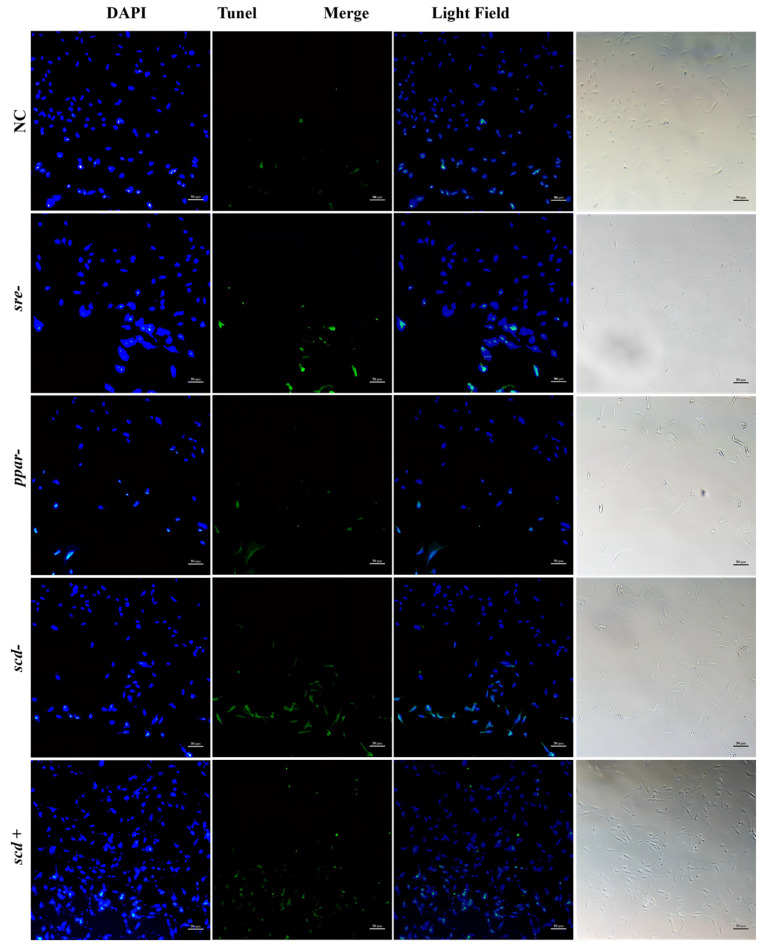
Apoptosis in silver pomfret hepatocytes after gene knockdown or overexpression at 10 °C (TUNEL-DAPI double staining, 10×).

Figure 9 presents the results at 10 °C. In the NC group, the cell number decreased compared with those at 26 °C and 18 °C, but green fluorescence signals were weaker, indicating a low level of apoptosis (~5% of total cells). The RNAi-*srebp* group also showed reduced cell numbers, with stronger green fluorescence than the NC group, corresponding to increased apoptosis (~20% of total cells). In the RNAi-*pparγ* group, cell numbers decreased significantly compared with those at higher temperatures, and fluorescence intensity was higher than in the NC group, representing about 10% apoptotic cells. The RNAi-*scd1* group also displayed markedly reduced cell numbers at 10 °C, accompanied by significantly increased apoptosis (~20% of total cells), which was higher than that observed in the NC group at the same temperature. Interestingly, cell numbers increased in the *scd1* overexpression group, with a rise in apoptotic signals (~20% of total cells), albeit with relatively weak fluorescence intensity.

## 4. Discussion

Fish initiate a series of adaptive processes in response to chronic low-temperature stress, in which genes and proteins involved in lipid metabolism play critical roles. Lipid metabolites not only serve as energy sources but also act as key signaling molecules in maintaining energy homeostasis and immune balance [25]. Stearoyl-CoA desaturase 1 (SCD1), a key enzyme in fatty acid desaturation, not only regulates fatty acid composition to enhance cold resistance but also modulates the ratio of saturated to unsaturated fatty acids in membrane phospholipids, thereby increasing membrane fluidity and improving cell survival under low-temperature conditions [16,26]. Gene expression exhibits complex spatiotemporal specificity, varying significantly across different tissues, and investigating these spatiotemporal expression patterns is essential for understanding functional regulation. However, the expression patterns of lipid metabolism-related genes and proteins across different tissues during cold adaptation in fish remain incompletely understood.

### 4.1. Tissue-Specific Expression Patterns of Lipid Metabolism Genes and Their Physiological Significance

Based on KEGG pathway, GSEA, and PPI network analyses of previous transcriptomic data, this study identified ten genes with potential upstream/downstream or interactive relationships with *scd1*. Quantitative analysis of these genes across different tissues revealed significant differences in tissue responses to low temperature, reflecting the distinct physiological roles of each tissue during cold adaptation.

Liver and heart: core organs for early energy mobilization. The liver exhibited the earliest and most robust activation of fatty acid catabolism-related genes (*cact*, *pparα*, *pparγ*) during the early cooling phase (16–12 °C) (Figure 1). This rapid response aligns with the liver’s central role in systemic energy homeostasis, prioritizing substrate mobilization to sustain vital functions under thermal stress [17,27,28]. Similar temporal patterns have been observed in common carp (*Cyprinus carpio*) under cold exposure [29], suggesting a conserved hepatic strategy for acute cold adaptation across teleosts. Notably, the sharp downregulation of these genes in the late cooling phase may reflect a shift from active catabolism to energy conservation, possibly mediated by negative feedback mechanisms to prevent excessive metabolic exhaustion. The heart, as the driving organ of the circulatory system, also showed upregulation of lipid metabolism-related genes (*pparα*, *pparγ*, *scd1*, *mct*) during the early cooling phase (Figure 2), consistent with reports that low temperatures increase cardiac contraction frequency and promote fatty acid metabolism to provide more energy [30].

Gill: membrane lipid remodeling in the mid-to-late stage. Gill tissue showed high expression of *pi3k*, *p450*, *cact*, *srebp*, and *scd1* during the mid-to-late stage of cold stress (10 °C) (Figure 3). Studies indicate that *pi3k*, regulated by insulin and leptin, influences the transcriptional regulator SREBP to activate downstream *scd1* expression, affecting triglyceride synthesis [31]. Cytochrome P450 is involved in fatty acid homeostasis [32]. SREBP, a transcription factor located on the endoplasmic reticulum membrane, binds to the promoter region of *scd1* to regulate its transcription [33,34], thereby increasing the proportion of unsaturated fatty acids. We hypothesize that under low temperatures, silver pomfret gill tissue increases the expression of *pi3k* and *p450* to activate the downstream transcription factor SREBP, initiating *scd1* expression. This process likely helps mitigate tissue and cellular damage caused by cold stress by adjusting the unsaturated fatty acid ratio [25] and enhancing cell membrane fluidity. Notably, the magnitude of upregulation in gill tissue (approximately 10-fold) was much lower than that in the liver (thousands-fold), which may reflect that the gill is not a primary site for lipid metabolism, with its lipid metabolic regulation more focused on maintaining barrier function and membrane stability.

Muscle, kidney, and brain: diverse response patterns. Muscle tissue exhibited two distinct upregulation events during cooling (at 14 °C and 6 °C) (Figure S2), which may relate to the dual roles of muscle in energy storage and thermogenesis. In the kidney, most genes were significantly upregulated during the later stages of cooling (Figure S3), likely reflecting adaptive adjustments in excretion and osmoregulation under low temperatures. Brain tissue showed peak gene expression at both the early (16 °C) and late (6 °C) stages of cooling (Figure S4), suggesting a two-phase response mechanism in the nervous system—“early sensing” followed by “late protection.”

Integrating the expression profiles across seven tissues, we observed a clear “temporal division of labor” during chronic cold adaptation in silver pomfret (Figure 1, Figure 2, Figure 3 and Figure 4 and S2–S4): the liver and heart respond rapidly during the early cooling phase (16–12 °C), prioritizing fatty acid mobilization for energy supply; the gill initiates membrane lipid remodeling during the mid-to-late stage (10 °C) to maintain barrier function; the kidney and brain show significant responses during the final stage (6 °C), potentially involving excretion homeostasis and neuroprotection; and muscle tissue exhibits two distinct upregulation events during both the early (14 °C) and late (6 °C) cooling phases, reflecting its dual role in energy storage and thermogenesis. This ordered temporal coordination reflects the organism’s prioritization strategy in response to environmental stress: first ensuring energy supply to core organs (liver, heart), then adjusting structural adaptations in tissues in direct contact with the environment (gill), then optimizing functional homeostasis in supporting organs (kidney, brain), while muscle serves as a versatile tissue contributing to both energy mobilization and thermal regulation. This multi-level spatiotemporal coordination may represent an efficient adaptive strategy evolved by teleosts for long-term cold adaptation.

### 4.2. Molecular Mechanisms of scd1 in Regulating Cell Apoptosis

SCD1 is a key regulator of cell proliferation, survival, and cancer transformation, influencing both autophagy and apoptosis. In silver pomfret hepatocytes, RNAi-mediated knockdown of *scd1* significantly reduced its gene expression, with lower expression observed at lower temperatures (Figure 6E). Correspondingly, Scd1 protein levels decreased significantly at 26 °C and 10 °C, accompanied by a significant increase in apoptosis (Figure 7, Figure 8 and Figure 9). Conversely, overexpression of *scd1* led to a significant increase in gene expression (Figure 6H), consistently low levels of apoptosis, and an increase in cell number at 10 °C (Figure 9). These findings indicate that *scd1* directly regulates apoptosis—its knockdown promotes apoptosis, while its overexpression inhibits it [35]. Similar results have been observed in mammalian cell lines [36,37].

The mechanisms by which SCD1 influences apoptosis may include two aspects: (1) SCD1 alters membrane fluidity by regulating the ratio of saturated to unsaturated fatty acids in the cell membrane; its inhibition can lead to altered membrane permeability, causing outflow of intracellular contents and triggering apoptosis [38]. (2) SCD1 regulates fatty acid saturation, modifying the lipid environment of the endoplasmic reticulum membrane and affecting the proper folding of newly synthesized proteins; misfolded proteins may trigger the unfolded protein response (UPR), and sustained ER stress can lead to apoptosis [39]. Notably, the lack of significant decrease in protein expression or increase in apoptosis at 18 °C (Figure 6I and Figure 8) may be because this temperature does not pose a survival challenge to silver pomfret hepatocytes—cell viability assays also indicated higher survival rates at 18 °C than at 26 °C (Figure 5), suggesting this temperature may be more favorable for cell survival.

### 4.3. Regulatory Roles of Upstream Factors srebp and pparγ on scd1

*Srebp* and *pparγ* are among the most frequently reported upstream regulators of *scd1* in the literature, capable of binding to its promoter region to control transcription [17]. The results of this study confirm this regulatory relationship: *srebp* knockdown significantly reduced *scd1* gene expression at 26 °C, 18 °C, and 10 °C (Figure 6F), but at the protein level, inhibition was observed only at 26 °C, with no significant effects at 18 °C and 10 °C (Figure 6I). This may be due to delays in protein translation and post-translational modifications under low-temperature conditions. Notably, *srebp* knockdown did not significantly alter apoptosis levels in hepatocytes, suggesting the existence of compensatory mechanisms.

In contrast, *pparγ* knockdown led to significant increases in both *scd1* gene and protein expression at 18 °C (Figure 6G,I), accompanied by apoptosis levels significantly lower than those in the NC group at the same temperature. This suggests a potential negative regulatory relationship between *pparγ* and *scd1*. This finding is consistent with studies in mice, where targeted disruption of an SCD1 isoform significantly increased ‘*pparγ-1α*’ expression [40]. We hypothesize that at specific temperatures (18 °C), *pparγ* may act as a negative regulator of *scd1*, and this negative feedback mechanism may help fine-tune fatty acid desaturation levels to avoid metabolic imbalance caused by over-adaptation. However, the specific regulatory mechanisms of *scd1* under low-temperature stress in fish remain unreported and require further validation through protein interaction assays or chromatin immunoprecipitation experiments.

### 4.4. Limitations and Future Perspectives

In this study, we focused on ten key genes closely associated with lipid metabolism at the tissue level, covering major metabolic processes including fatty acid synthesis, desaturation, transport, and oxidative catabolism. However, lipid metabolism is a highly complex physiological process regulated by multiple layers of control, involving the coordinated action of numerous genes, transcription factors, and metabolic enzymes. Therefore, expression analysis based on a limited set of targeted genes may not fully capture the comprehensive landscape of lipid metabolic reprogramming under cold stress. Future studies integrating multi-omics approaches such as transcriptomics and metabolomics will be valuable for systematically dissecting the global regulatory networks underlying lipid metabolism during cold adaptation in silver pomfret. Furthermore, the negative regulatory relationship between *pparγ* and *scd1* observed at specific temperatures, as well as the molecular mechanisms underlying the temporal division of labor among tissues, warrant further validation through promoter activity assays, ChIP-qPCR, and protein interaction experiments. These investigations will provide important theoretical foundations for understanding the molecular mechanisms of cold adaptation in fish and for breeding cold-resistant strains.

## 5. Conclusions

This study elucidates the adaptive mechanisms of silver pomfret in response to low-temperature stress, highlighting the pivotal role of the key lipid metabolism gene *scd1* and its regulatory network. The results demonstrate that *scd1* directly regulates cell survival and apoptosis under cold conditions, and its expression is intricately modulated by upstream regulators *srebp* (positive regulation) and *pparγ* (showing potential negative feedback at specific temperatures). Different tissues exhibited a temporal division of labor: the liver and heart rapidly activated lipid catabolism genes for energy supply during the early cooling phase, while gill tissue upregulated the *pi3k*/*p450*/*srebp*/*scd1* pathway in the mid-to-late stages to remodel membrane lipids against prolonged cold stress. These findings identify *scd1* and its regulatory genes as candidate molecular markers for cold tolerance, which can be utilized in marker-assisted selection to accelerate the breeding of cold-resistant silver pomfret strains. Furthermore, the tissue-specific temporal response patterns provide a theoretical basis for optimizing aquaculture management strategies, such as adjusting feeding regimes or water temperature control during different cold exposure phases. Future research should focus on validating the regulatory interactions identified in this study through techniques such as ChIP-qPCR and dual-luciferase reporter assays, and on integrating multi-omics approaches to comprehensively map the lipid metabolic network underlying cold adaptation.

## Figures and Tables

**Table 1:** Genes and specific primers for RT-qPCR.

Gene Names	Reference Sequence IDs	Primer 5′–3′	Products Length	T_m_
*scd1*	evm.TU.scaffold27.368	AAATGCTGCTATCACTGCTACG	248 bp	58 °C
		TCCCCTTTGACTACGCCAC		
*mct*	evm.TU.scaffold63.358	CTGGTAGTAGTGGCATTGGGA	284 bp	58 °C
		TTGCTTCGGCTCTGTTCG		
*cact*	evm.TU.scaffold17.553	TCCTGGCTTTCCACCTATCC	118 bp	57 °C
		GCAATCGTTTGGTCCTTCCT		
*pi3k*	evm.TU.scafold545.88	AGCGAGGAAAAGTTCAAGCA	163 bp	57 °C
		TCCCCAAGATGTGACCGA		
*stc*	evm.TU.scaffold224.190	GATTCAAAGTTGCCTGGTGG	109 bp	58 °C
		CAGAGTGAGACAGATGTGGTGC		
*ltp*	evm.TU.scaffold131.173	TCGTTGGTGAACTACGCTGTG	124 bp	59 °C
		TCTGGAAGAGTCCTGCCTGAT		
*p450*	evm.TU.scafold14.571	AGAAAACCTCCACCAGACCTT	187 bp	57 °C
		GTAGCCCGACTCAAACATCAC		
*srebp*	evm.TU.scafold88861.366	TACCGCTCCTCCATCAACG	235 bp	58 °C
		TCTTCACATCAGCAGGTCCAT		
*pparα*	evm.TU.scaffold545.187	CATCGCACTGGACACCTTG	196 bp	58 °C
		GGATGGTCCTCCTGAAGAAAC		
*pparγ*	evm.TU.scaffold17.990	GACAGCCGTTGTTGGATGAC	143 bp	57 °C
		GCTTGGGTGTTGTAGGAGGA		

**Table 2:** Sequences of siRNA.

Gene Name	Sense (5′–3′)	Antisense (5′–3′)
*srebp*-1	GUGGGCAUGUUGGACAAUATT	UAUUGUCCAACAUGCCCACTT
*srebp*-2	GGAGGACUGUUUGAUAACUTT	AGUUAUCAAACAGUCCUCCTT
*srebp*-3	GCCAGCAAAUGAUCAUCAATT	UUGAUGAUCAUUUGCUGGCTT
*pparγ*-1	GCCCAAGUUUGAAGUUUGUTT	ACAAACUUCAAACUUGGGCTT
*pparγ*-2	CGGGAGAUCACAUGCAAAUTT	AUUUGCAUGUGAUCUCCCGTT
*pparγ*-3	GGCCUCUGUAUGGAACUAUTT	AUAGUUCCAUACAGAGGCCTT
*scd1*-1	CAGGGUUCAUCACAAAUAUTT	AUAUUUGUGAUGAACCCUGTT
*scd1*-2	GCGAAGGAUUUCACAAUUATT	UAAUUGUGAAAUCCUUCGCTT
*scd1*-3	CCUCCCACAAUCAUCGUAUTT	AUACGAUGAUUGUGGGAGGTT

## Data Availability

The transcriptomic data generated in this study have been deposited in the NCBI database under accession number PRJAN783750. The reference sequence IDs for the ten lipid metabolism-related genes analyzed in this study are provided in Table 1. These data are openly available from the NCBI repository (https://www.ncbi.nlm.nih.gov/, accessed on 12 January 2025).

## Supplementary Materials

The following supporting information can be downloaded at: https://www.mdpi.com/article/10.3390/ani16081196/s1, Figure S1: Schematic of fish and cell experiments. Figure S2: Variation in lipid metabolism DEGs in silver pomfret muscle under low temperature (*n* = 3). X-axis = Temperature; Y-axis = Fold change (2^−ΔΔCt^). Figure S3: Variation in lipid metabolism DEGs in silver pomfret kidney under low temperature (*n* = 3). X-axis = Temperature; Y-axis = Fold change (2^−ΔΔCt^). Figure S4: Variation in lipid metabolism DEGs in silver pomfret brain under low temperature (*n* = 3). X-axis = Temperature; Y-axis = Fold change (2^−ΔΔCt^). Figure S5: Cloned sequence of scd1.
