# Supplementary figures and images for: Cold Stress Induces Tissue-Specific Lipid Metabolic Responses and Scd1-Mediated Hepatic Apoptosis in Silver Pomfret
Source: Animals (Basel). 2026 Apr 14;16(8):1196. doi: 10.3390/ani16081196 (PMC13113318; doi:10.3390/ani16081196)

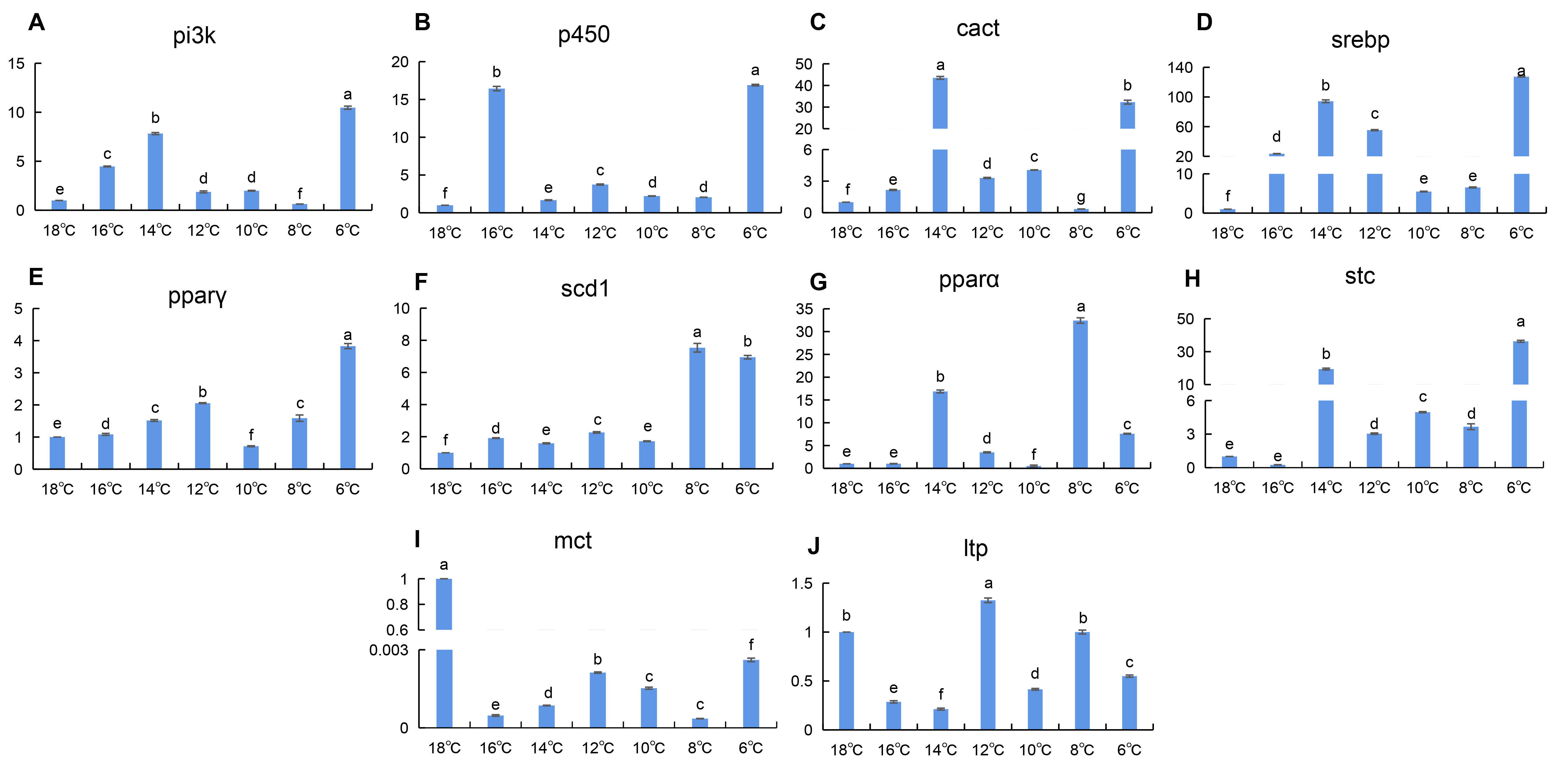

Supplement: Supplementary file 1 [file animals-16-01196-s001.zip › Figure S2.png]

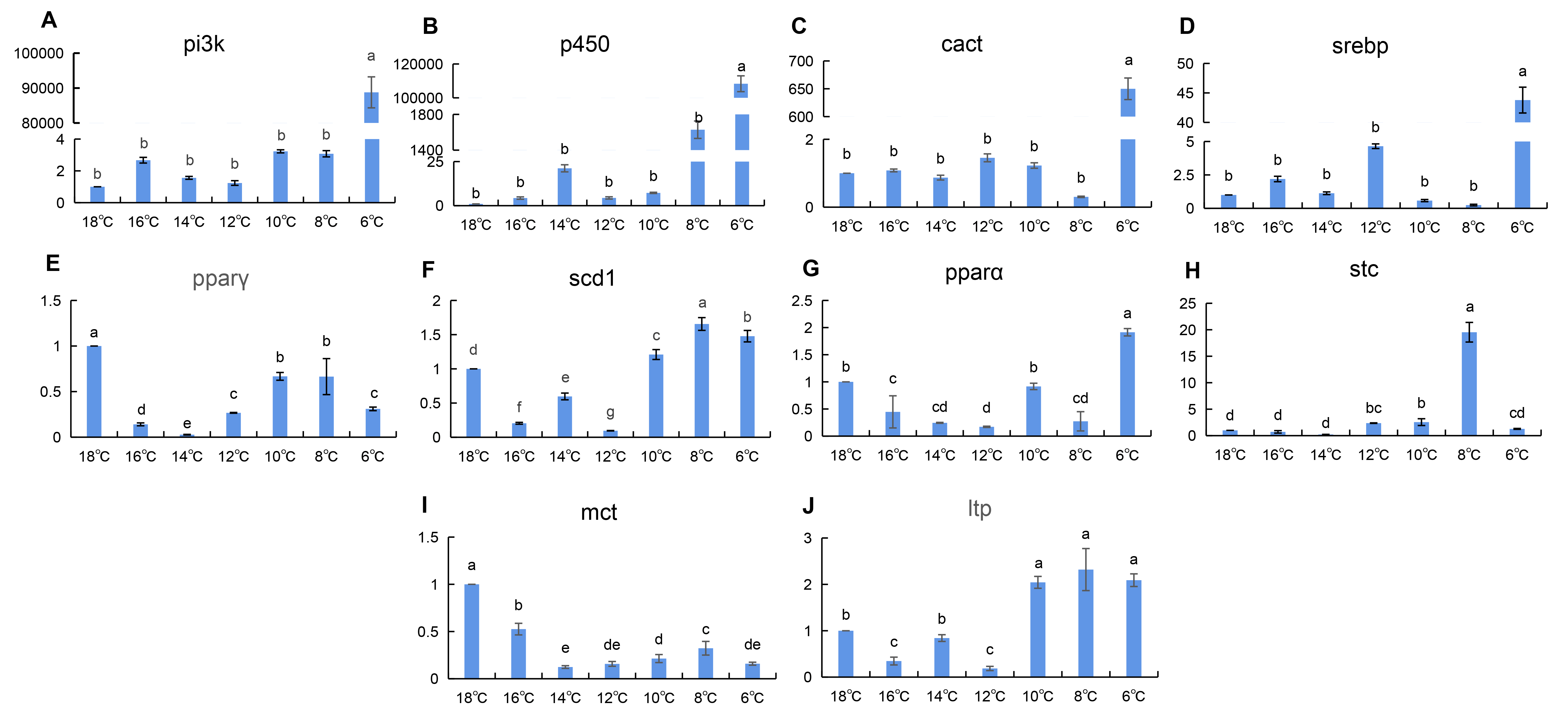

Supplement: Supplementary file 1 [file animals-16-01196-s001.zip › Figure S3.png]

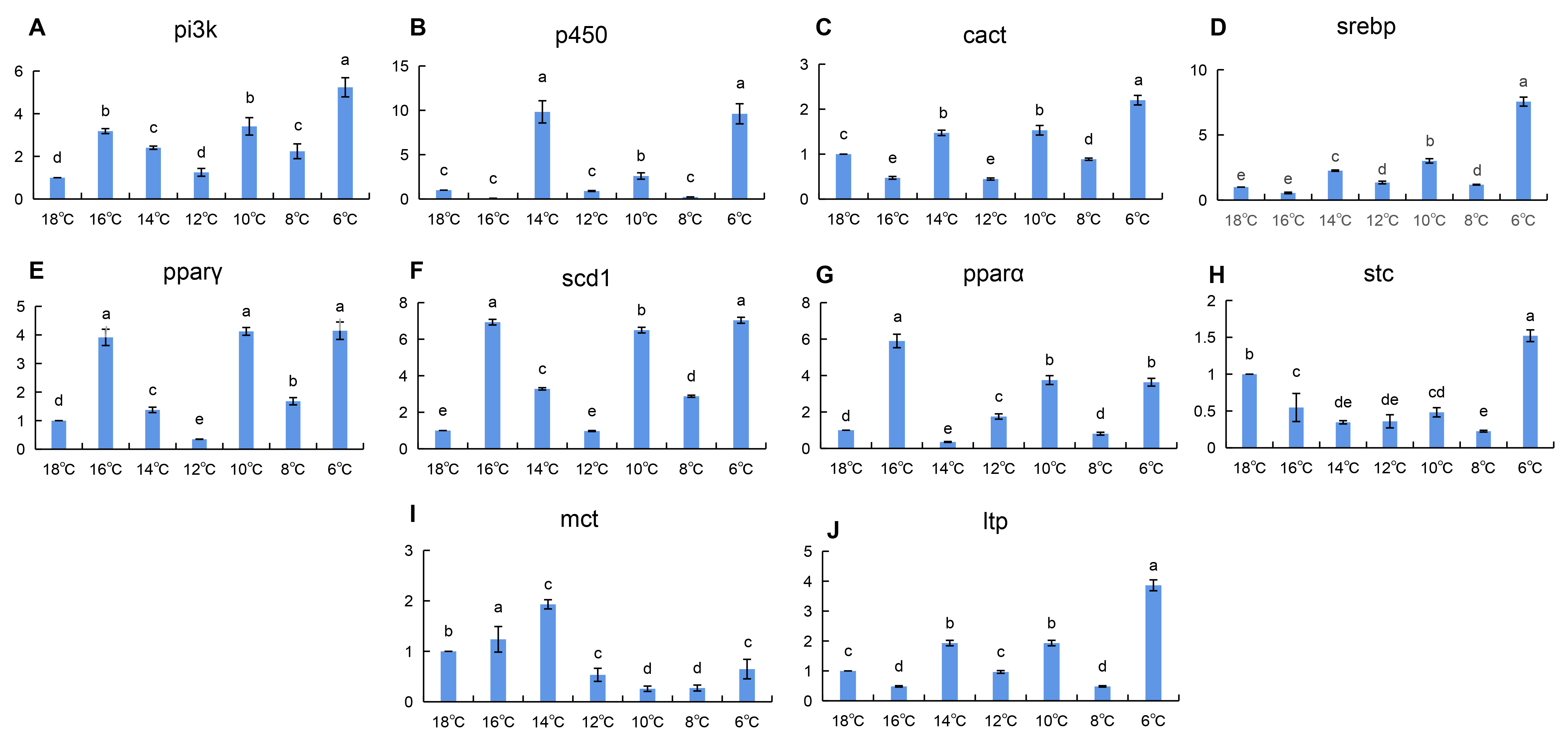

Supplement: Supplementary file 1 [file animals-16-01196-s001.zip › Figure S4.png]

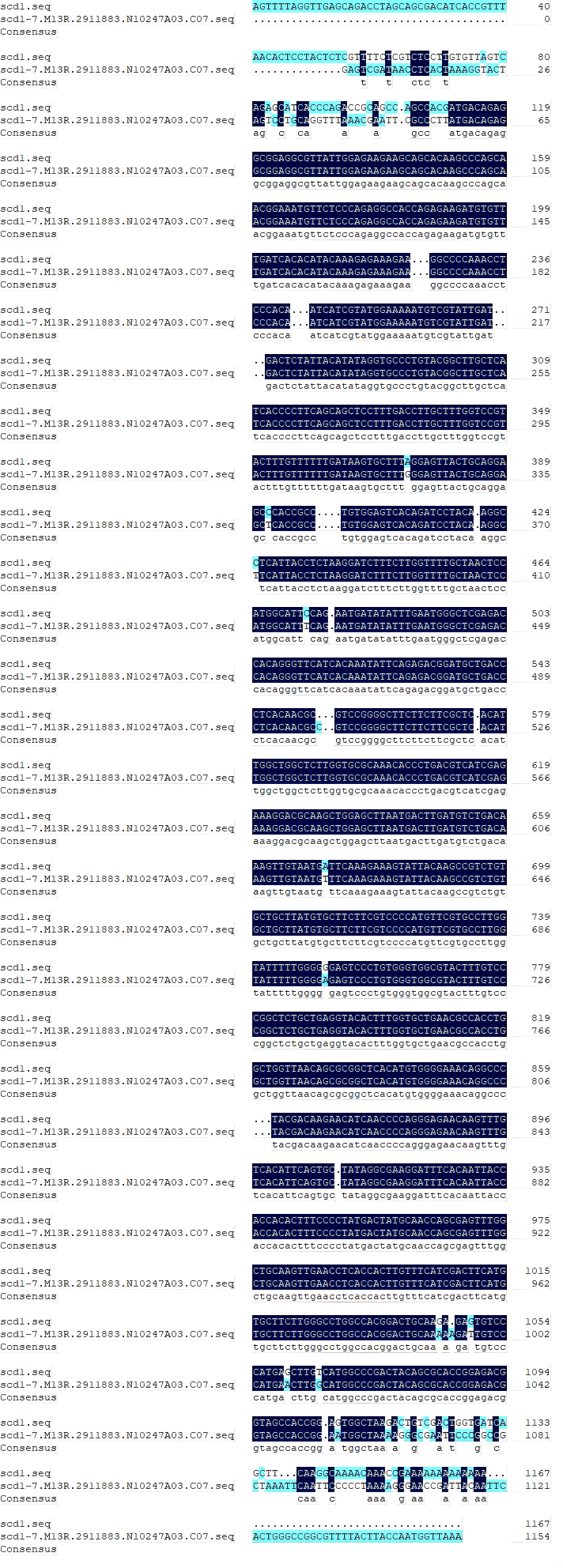

Supplement: Supplementary file 1 [file animals-16-01196-s001.zip › Figure S5.png]
